# circROBO1 promotes prostate cancer growth and enzalutamide resistance via accelerating glycolysis

**DOI:** 10.7150/jca.86940

**Published:** 2023-08-21

**Authors:** Zhigang Zhou, Jing Qin, Cailu Song, Tao Wu, Qiang Quan, Yan Zhang, Yani Zou, Lingrui Liu, Hailin Tang, Jianfu Zhao

**Affiliations:** 1 Research Center of Cancer Diagnosis and Therapy, Department of Oncology, The First Affiliated Hospital of Jinan University, Guangzhou 510632, China.; 2 Changde Hospital, Xiangya School of Medicine, Central South University, Changde, 415003, China.; 3 State Key Laboratory of Oncology in South China, Sun Yat-sen University Cancer Center, Guangzhou, 510060, China.; 4 The First People's Hospital of Fuyang City, Fuyang, 236012, China.

**Keywords:** circROBO1, circular RNAs, PGK1, enzalutamide resistance, prostate cancer

## Abstract

**Background and aim:** As non-coding RNAs, circular RNAs (circRNAs) contribute to the progression of malignancies by regulating various biological processes. In prostate cancer, however, there is still a lack of understanding regarding the potential molecular pathways and roles of circRNAs.

**Methods:** Loss-off function experiments were performed to investigate the potential biological function of circRNA in the progression of prostate cancer. Western blot, qRT-PCR, and IHC assay were used to examine the expression level of different genes or circRNAs. Further molecular biology experiments were conducted to uncover the molecular mechanism underlying circRNA in prostate cancer using dual luciferase reporter and RNA immunoprecipitation (RIP) assays.

**Results:** A novel circRNA (hsa_circ_0124696, named circROBO1) was identified as a significantly upregulated circRNA in both prostate cancer cells and tissues. Suppression of circROBO1 significantly attenuated the proliferation of prostate cancer cells. In addition, we found that the knockdown of circROBO1 remarkably increased the sensitivity of prostate cancer to enzalutamide treatment. A deceleration in glycolysis rate was observed after inhibition of circROBO1, which could suppress prostate cancer growth and overcome resistance to enzalutamide. Our results revealed that circROBO1 promotes prostate cancer growth and enzalutamide resistance via accelerating glycolysis.

**Conclusion:** Our study identified the biological role of the circROBO1-miR-556-5p-PGK1 axis in the growth and enzalutamide resistance of prostate cancer, which is the potential therapeutic target of prostate cancer.

## Introduction

Prostate cancer is the most common malignancies in men worldwide, with a high incidence and mortality rate 1. Prostate cancer is classified into different types based on the histological characteristics of the tumor, including adenocarcinoma, small cell carcinoma, and neuroendocrine carcinoma 2. The prognosis of prostate cancer varies depending on the stage and grade of the tumor, with early-stage and low-grade tumors having a better prognosis than advanced-stage and high-grade tumors 3, 4. The treatment of prostate cancer includes surgery, radiation therapy, chemotherapy, and hormone therapy. Hormone therapy, also known as androgen deprivation therapy (ADT), is a common treatment option for advanced or metastatic prostate cancer 5, 6. ADT works by reducing the levels of androgens, such as testosterone, in the body, which can slow down the growth of prostate cancer cells 7.

Enzalutamide, a second-generation antiandrogen, has been approved for the treatment of metastatic castration-resistant prostate cancer (mCRPC) and has shown remarkable efficacy in clinical trials. Enzalutamide exerts its therapeutic effect by inhibiting the androgen receptor (AR) signaling pathway, which plays a crucial role in the development and progression of prostate cancer. Enzalutamide has been shown to prolong overall survival and delay disease progression in patients with mCRPC in several clinical trials, including the AFFIRM trial and the PREVAIL trial 8, 9. However, the emergence of enzalutamide resistance has become a major challenge in the management of mCRPC. The clinical statistics show that approximately 20-40% of patients with mCRPC do not respond to enzalutamide, and the majority of responders eventually develop resistance after a median time of 9-15 months 10, 11. Therefore, it is curial investigate the molecular pathogenesis of prostate cancer and to develop novel therapeutic strategies to overcome the enzalutamide resistance.

Circular RNAs (circRNAs) are a class of non-coding RNAs (ncRNAs) that have recently gained attention due to their potential roles in various biological processes 12. Unlike other linear RNAs, circular RNAs have a covalently closed loop structure, which increases their stability and resistance to degradation 13. Additionally, circRNAs are highly conserved and tissue-specific, and their expression levels are regulated in a cell- and developmental stage-specific manner 14. These unique features of circRNAs make them attractive candidates for biomarkers and therapeutic targets in various diseases. There have been many studies showing that circRNAs are involved in the regulation of various diseases, including diabetes, neurological disorders, autoimmune disorders, heart failures, and cancers 15, 16. Circular RNA sponge for miR-7 (ciRS-7) is a circular RNA molecule that has been recently identified as a key regulator of cancer progression. It acts as a competitive endogenous RNA (ceRNA) by binding to miR-7 and preventing its interaction with target mRNAs. This results in the upregulation of miR-7 target genes, which are involved in various cellular processes such as proliferation, apoptosis, and migration 17-21. Studies have shown that circHIPK3 acts as a sponge for microRNAs, thereby regulating the expression of downstream target genes. For example, circHIPK3 has been found to sponge miR-124, leading to increased expression of their target genes, which are involved in cell proliferation and apoptosis 22. In addition, circEZH2 has been found to play a crucial role in regulating tumor progression. The expression level of circEZH2 has been shown to be upregulated in various types of cancers, including glioblastoma, breast cancer, and some other tumors. It is revealed that EZH2-92aa, encoded by circEZH2, inhibits surface NKG2D ligand binding in glioblastoma cells 23. By enhancing the epithelial-mesenchymal transition, the FUS/circEZH2/KLF5 feedback loop promotes CXCR4-induced liver metastasis of breast cancer 24. In addition, the researchers investigated the potential application of circRNA in glioma tumorigenesis, and demonstrate the inhibitory effects of isoliquiritigenin on circ0030018 via the miR-1236/HER2 signaling pathway 25. In our recent study, we investigated the role of circROBO1 in regulating the liver metastasis of breast cancer through a feedback loop involving KLF5 and FUS, which inhibits the selective autophagy of afadin 26. Nevertheless, little is known about how circRNAs might contribute to prostate cancer and their mechanisms.

In this study, we identified a novel circRNA (hsa_circ_0124696, named circROBO1) as a frequently upregulated circRNA in both prostate cancer cells and tissues. To investigate how circROBO1 may contribute to prostate cancer progression, loss-off function experiments were performed. The inhibition of circROBO1 significantly decreased the proliferation of prostate cancer cells. In addition, we found that the knockdown of circROBO1 remarkably increased the sensitivity of prostate cancer to enzalutamide treatment. A deceleration in glycolysis rate was observed after inhibition of circROBO1, which could suppress prostate cancer growth and overcome resistance to enzalutamide. We performed further molecular biology experiments using a dual luciferase reporter assay and RNA immunoprecipitation assays to obtain further insight into the molecular mechanism behind circROBO1 in prostate cancer. Our results revealed that circROBO1 promotes prostate cancer growth and enzalutamide resistance via accelerating glycolysis. Generally, our study identified the biological role of the circROBO1-miR-556-5p-PGK1 axis in the growth and enzalutamide resistance of prostate cancer, which could be an effective therapeutic target.

## Methods and materials

### Clinical tissue collection

We obtained fresh nearby normal prostate tissues and primary prostate cancer samples from the First Affiliated Hospital of Jinan University. Only patients with early-stage prostate cancer were included in the study. Patients with incomplete clinicopathological information or pathologically unconfirmed prostate cancer were excluded. The detailed clinic parameters of the enrolled patients in this study are displayed (Supplementary Table 1). This study was approved by the Ethics Committee of the the First Affiliated Hospital of Jinan University. Written informed consent was collected from all prostate cancer patients before participation in this study.

### Cell line preserve and culture

The cell lines used in this study were all purchased from the ATCC (WPMY1, C4-2B, LNCaP, 22RV1, VCaP, DU145, PC-3, and HEK293T). Culture of cell lines was performed according to instructions provided by the supplier. WPMY1, 22RV1, VCaP, and HEK293T cell lines were cultured in DMEM + 10% FBS + 1% P/S. C4-2B, LNCaP, and DU145 cell lines were cultured in RPMI1640 + 10% FBS + 1% P/S. Cells were cultured at 37 ° C in an incubator containing 5% CO_2_. The authenticity of cells was occasionally verified through the use of DNA fingerprinting.

### Nuclear and cytoplasmic fractionation

Nuclear and cytoplasmic RNA from prostate cancer cells were separated by the PARIS Kit (Invitrogen, CA, USA) according to the protocols.

### RT-qPCR analysis

Total cellular RNA was extracted using the TRIzol reagent (Invitrogen, USA). In this study, we used a SYBR Premix Ex Taq Kit from Takara (Japan) for qRT-PCR. The sequences of the primers used in this study is displayed in Supplementary Table 2.

### Western blot analysis

The cells were lysed using RIPA lysis and PMSF to isolate the total protein. Proteins were separated on SDS-PAGE gels in each well. A PVDF membrane was then used to move the protein for two hours at 300 mA for two hours. As a next step, we blocked the membranes with 5% skim milk powder. Each antibody was incubated overnight at 4°C on the membrane, followed by one hour at room temperature with the specific secondary antibody. The protein bands were eventually detected by chemiluminescence. The anti-PGK1 (1:1000, CST, 63536S, USA) and anti-beta-actin antibody (1:1000, CST, 4970S, USA) are used to detect certain protein.

### Actinomycin D assay

Actinomycin D was used to degrade the linear mRNA transcription of C4-2B prostate cancer cells for 0, 8, 16, and 24 hours. By using qPCR-analysis, the linear ROBO1 mRNA and circular circROBO1 mRNAs were detected in C4-2B cells at certain times.

### RNase R digestion assay

After 5 ug of the extracted total RNA from C4-2B prostate cancer cells were treated with RNase R (5 U / ug) or the blank control solution for 30 min at 37 ℃, a RT-qPCR analysis was performed on the resulting RNA solution to quantification.

### Lactate production and glucose consumption measurements

A glucose/glucose oxidase assay kit (Invitrogen, USA) was used to measure glucose consumption and lactate production. In order to normalize the data, the total amount of cellular proteins was taken into the account.

### CCK-8 assay

After digesting and resuspending the C4-2B and 22RV1 prostate cancer cells, they were resuspended in a suspension medium. Afterwards, 4000 cells per well were seeded into 96-well plates. T Cells were incubated at 37°C for each time period. After adding the CCK-8 solution (10 μl), incubation for an hour was performed before measuring the results.

### Colony formation assay

A total of 5×10^3^ cells were resuspended and seeded in the each well of a six-well plate. A 7-day incubation at 37°C is followed by methanol fixation and staining with 2.5% crystal violet, followed by an image analysis utilizing Image J software.

### Dual luciferase reporter assay

Synthesis of the sequences of circROBO1 or PGK1 3'-UTR that contain the wild-type (WT) or mutant-type (MT) miR-556-5p binding sites was performed. We then cloned wild-type or mutant sequences of circROBO1 or PGK1 3'-UTR into pmirGLO (Promega). A co-transfection of mimics-NC and mimics-miR-556-5p was then carried out in HEK293T cells with the corresponding plasmids. Following 48 hours of incubation, all co-transfections above were examined for Renilla and Firefly luciferase activity using the Dual Luciferase Reporter Assay Kit from Promega (Wisconsin, USA). Renilla activity was used as the internal reference.

### RNA immunoprecipitation (RIP)

Assays were conducted using Magna RIP kits (Millipore, MA, USA) according to the protocols provided. In RIP assays for Ago2 protein, anti-Ago2 antibodies were used. After RNA purification, circROBO1, PGK1, and miR-556-5p expression levels were assessed. C4-2B and 22RV1 prostate cancer cells were transfected with ms2-circROBO1 plasmid, ms2-circROBO1-mutant plasmid, and ms2bs-NC plasmid. RIP assays were performed following 72 hours of incubation. After purification of RNA complexes, the relative abundance of miR-556-5p was determined.

### Statistical analysis

The statistical analysis was performed using SPSS 22.0 (SPSS, USA). Data are reported as mean ± standard deviation (SD). Student's t test was used to compare two groups. Paired t test was used to compare the expression difference between normal and prostate cancer tissues. *P*<0.05 was considered as statistically significant.

## Results

### circROBO1 is upregulated in prostate cancer cells and tissues

**Figure 1:**
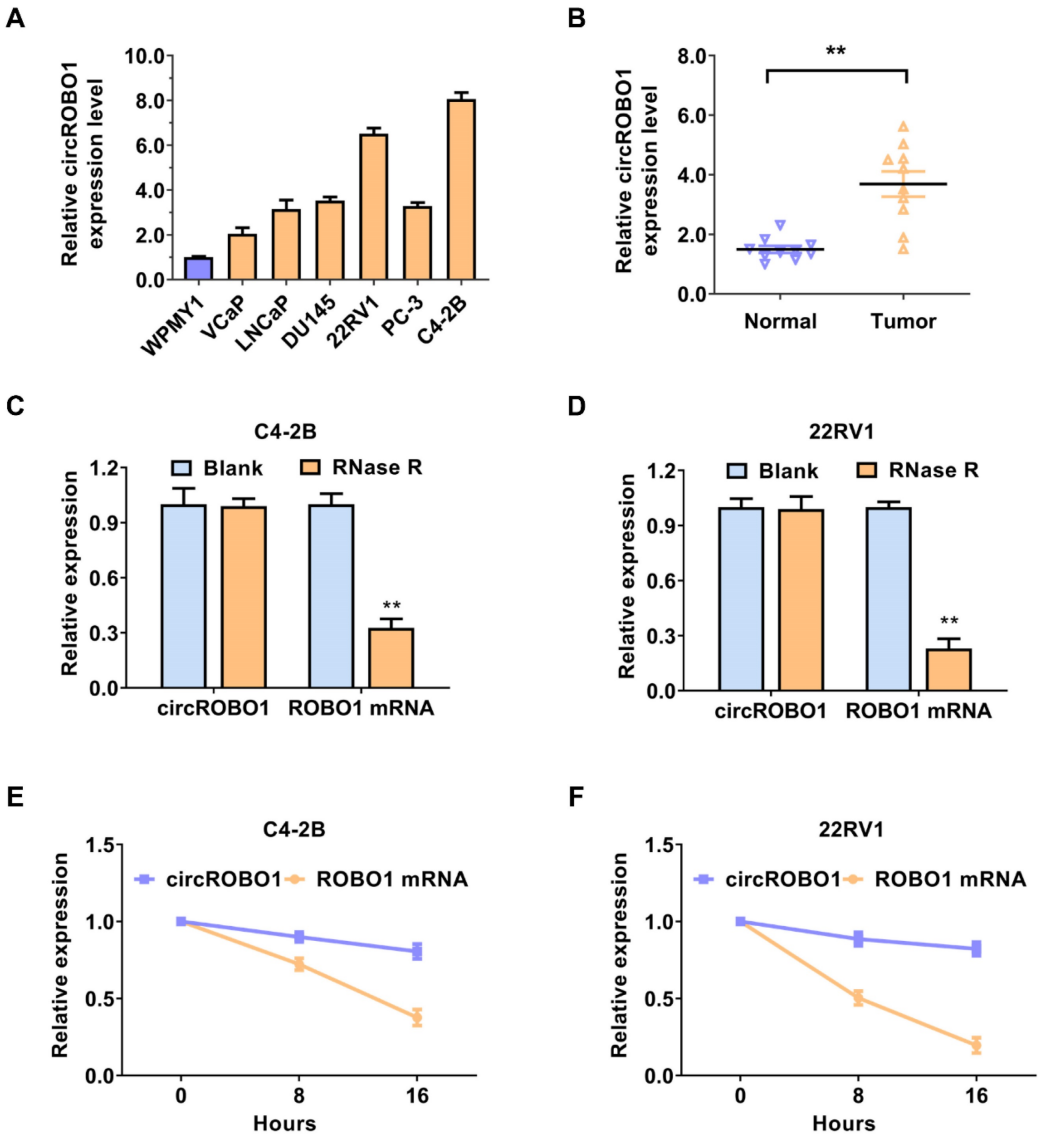
** circROBO1 is upregulated in prostate cancer cells and tissues. (A)** Comparison of the expression level of circROBO1 in normal WPMY1 cells and prostate cancer cells. **(B)** The expression level of the circROBO1 expression level in ten pairs of prostate cancer tissues and adjacent normal tissues, detected by qPCR analysis. **(C)** RNase R assays were used to examine circROBO1's circular feature in C4-2B and 22RV1 prostate cancer cell line. **(D)** Actinomycin D treated assays showed that circular ROBO1 transcripts were more stable than linear ROBO1 transcripts in C4-2B and 22RV1 prostate cancer cell line.

circROBO1 (hsa_circ_0124696) originates from exon 5, exon 6, exon 7, and exon 8 of the ROBO1 mRNA, with a back spliced junction site of exon 8 and exon 5. First, we detected the expression level of circROBO1 in prostate cancer lines using RT-qPCR analysis. CircROBO1 was upregulated in prostate cancer cell lines compared to normal cell WPMY1, especially in C4-2B and 22RV1 prostate cancer cell lines, determined by RT-qPCR analysis (Figure 1A). We used RT-qPCR to verify the expression level of the circROBO1 expression level in ten pairs of prostate cancer tissues and adjacent normal tissues (Figure 1B). The absolute expression levels of circROBO1 between normal and tumor prostate tissues is displayed in Supplementary Table 3. We found that circROBO1 was significantly upregulated in tumor tissue compared with their paired normal tissue (Figure 1B). A further investigation of circROBO1's circular structure and stability was conducted using actinomycin D and RNase R assays. In our study, we found that circROBO1 was resistant to RNase R (one kind of RNA exonuclease) (Figure 1C-D). Actinomycin D tests were conducted to determine circROBO1 and linear ROBO1 transcription half-lives in addition to evaluating the stability of circROBO1. We found that the circular ROBO1 mRNA has a longer half-life in actinomycin D assays than the linear ROBO1 mRNA (Figure 1E-F). These results verified that circROBO1 has the characteristics of circular RNA.

### circROBO1 promotes the glycolysis and enzalutamide resistance of prostate cancer cells

**Figure 2:**
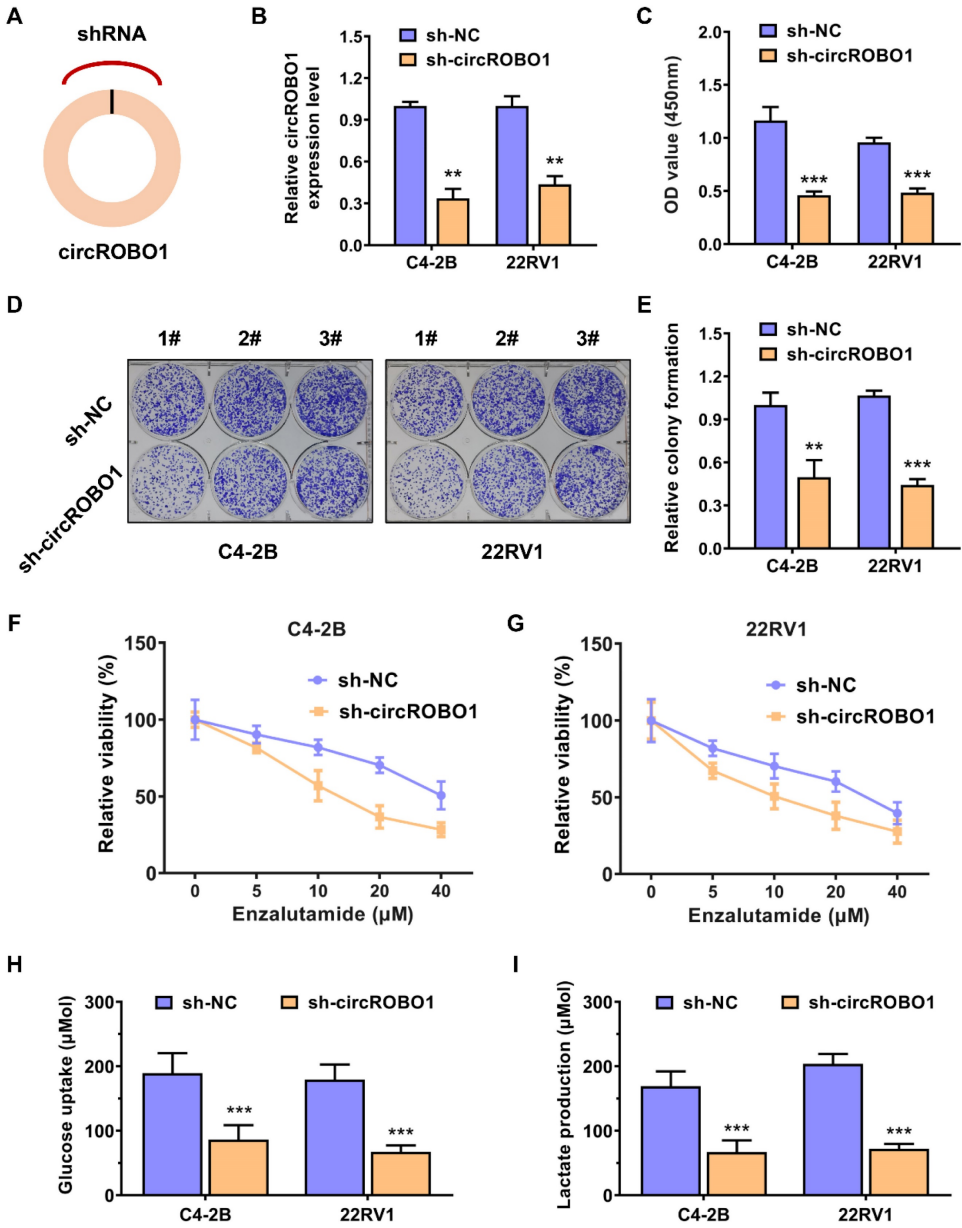
** circROBO1 promotes the glycolysis and enzalutamide resistance of prostate cancer cells. (A)** Short hairpin RNA (shRNA) targeting the back-splice sequence of circRNA were designed to investigate the function of circROBO1 in prostate cancer. **(B)** The efficacy of shRNA was determined in C4-2B and 22RV1 prostate cancer cell lines. **(C)** The proliferation of cells was evaluated using the CCK-8 assay in C4-2B and 22RV1 prostate cancer cell line. **(D)** The colony-formation ability of cells was evaluated using the Colony-formation assay in C4-2B and 22RV1 prostate cancer cell line.** (E)** A graph showing the statistical data for the colony-formation test. **(F-G)** Knockdown of circROBO1 significantly increased the sensitivity of C4-2B and 22RV1 prostate cancer cells to enzalutamide treatment. **(H-I)** circROBO1-induced glucose uptake and lactate production was found to accelerate glycolysis in C4-2B and 22RV1 prostate cancer cells.

Short hairpin RNA (shRNA) targeting the back-splice sequence of circRNA were designed to investigate the function of circROBO1 in prostate cancer (Figure 2A). The efficacy of shRNA was determined in C4-2B and 22RV1 prostate cancer cell lines (Figure 2B). Inhibition of circROBO1 significantly suppressed the proliferation rate of the C4-2B and 22RV1 prostate cancer cell lines in vitro, revealed by the CCK-8 assays (Figure 2C). Similarly, a colony-formation assay revealed similar results as a proliferation assay (Figure 2D). Knockdown of circROBO1 inhibited the colony-formation ability of the C4-2B and 22RV1 prostate cancer cells (Figure 2E). Targeting circROBO1 significantly increased the sensitivity of C4-2B and 22RV1 prostate cancer cells to enzalutamide treatment (Figure 2F-G). Glucose uptake and lactate production are two important rate-limiting steps in the biological process of tumor cell glycolysis. Also, circROBO1 could enhance glucose uptake and lactate production, thereby accelerating the glycolysis of C4-2B and 22RV1 prostate cancer cells (Figure 2H-I).

### circROBO1 acts as a sponge of miR-556-5p in prostate cancer

**Figure 3:**
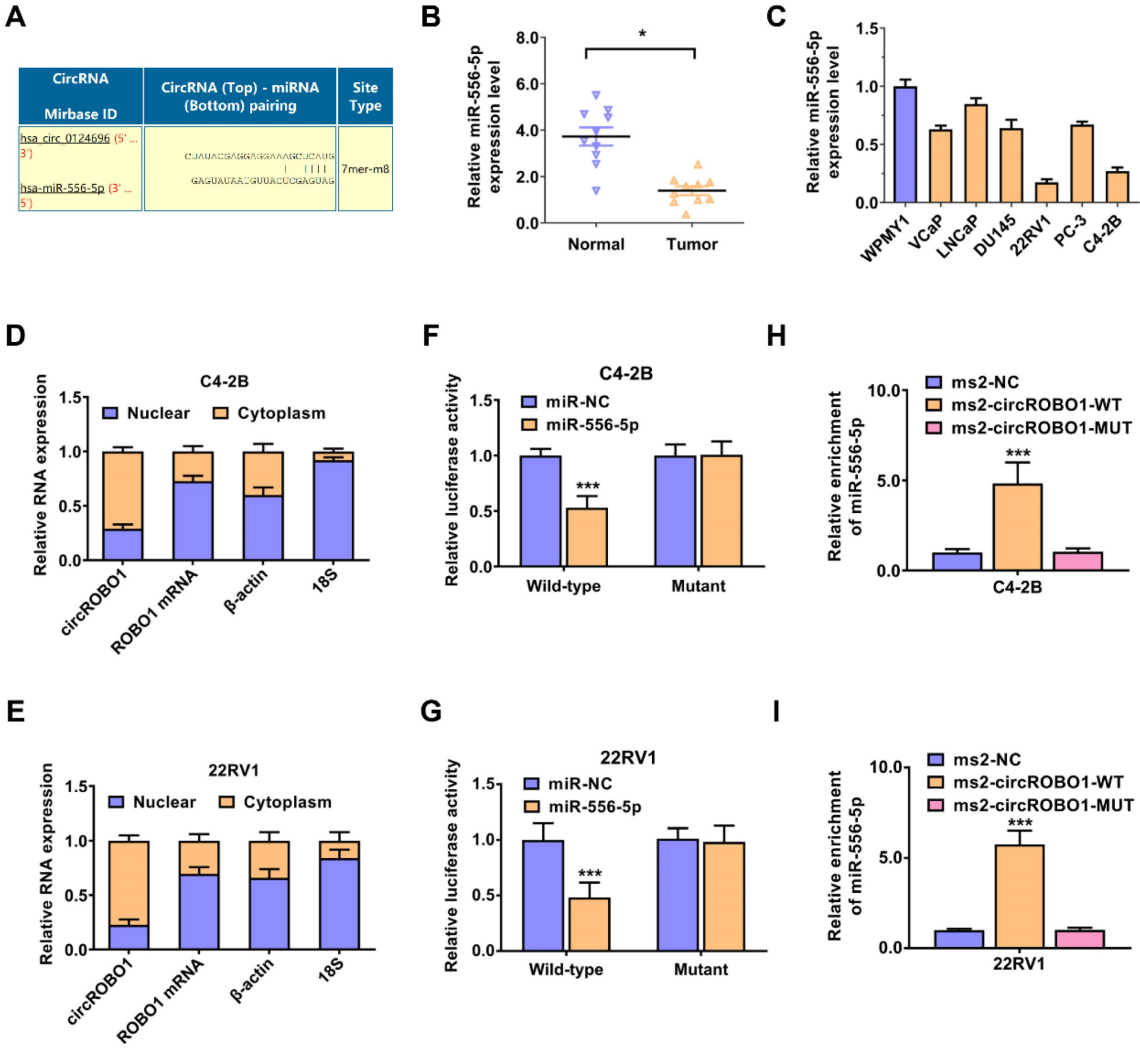
** circROBO1 acts as a sponge of miR-556-5p in prostate cancer. (A)** The predicted binding sites for miR-556-5p within circROBO1. **(B)** Comparison of the expression level of miR-556-5p in normal WPMY1 cells and prostate cancer cells.** (C)** The expression level of the miR-556-5p expression level in ten pairs of prostate cancer tissues and adjacent normal tissues, detected by qPCR analysis.** (D-E)** RT-qPCR analysis was performed on mRNA expression of 18S, ACTB, circROBO1 and ROBO1 in the nuclear and cytoplasmic fractions. **(F-G)** Dual luciferase reporter assay of C4-2B and 22RV1 prostate cancer cell lines transfected with miR-556-5p mimics and circROBO1 wild-type or mutant-type luciferase vectors. **(H-I)** The ms2-related RIP assay was performed by transfecting the ms2-circROBO1 plasmid, the ms2-circROBO1-mutant plasmid, or the Rluc-NC plasmid.

Then, we evaluated the potential interactions between circular RNAs and multiple miRNAs using the Circular RNA Interactome database. According to the predictions, miR-556-5p binds the circROBO1 sequence with possible interacting sites (Figure 3A). Next, we used RT-qPCR to verify the expression level of the miR-556-5p expression level in ten pairs of prostate cancer tissues and adjacent normal tissues (Figure 3B). We found that miR-556-5p was significantly downregulated in tumor tissue compared with their paired normal tissue (Figure 3B). Then, we also detected the expression level of miR-556-5p in prostate cancer lines using RT-qPCR analysis. miR-556-5p was extremely downregulated in prostate cancer cell lines compared to normal cell WPMY1, especially in C4-2B and 22RV1 prostate cancer cell lines, determined by RT-qPCR analysis (Figure 3C). To identify the subcellular localization of circROBO1, we performed RT-qPCR on the cytoplasmic and nuclear fractions of C4-2B and 22RV1 prostate cancer cells (Figure 3D). According to our results, circROBO1 is primarily located in the cytoplasm rather than in the nucleus (Figure 3E). To further confirm the interaction sites between circROBO1 and miR-556-5p, we constructed a wild-type and a mutant dual-luciferase reporter plasmids containing the predictive interacting sites between circROBO1 and miR-556-5p. Compared to the mutant group, miR-556-5p mimics significantly decreased the luciferase activity of wild-type group. In contrast, there was no detectable difference between the miR-556-5p mimics and control mimics group (Figure 3F-G). To confirm the direct interaction between circROBO1 and miR-556-5p, we next performed RIP assay to pulldown circROBO1 (Figure 3H-I). Our results showed that intracellular miR-556-5p were predominantly gathered in the ms2-circROBO1 plasmid overexpressed group (Figure 3H-I). These results indicated that circROBO1 could bind with directly miR-556-5p and act as a sponge of miR-556-5p in prostate cancer.

### Glycolysis regulating enzyme PGK1 is the downstream target of miR-556-5p in prostate cancer

**Figure 4:**
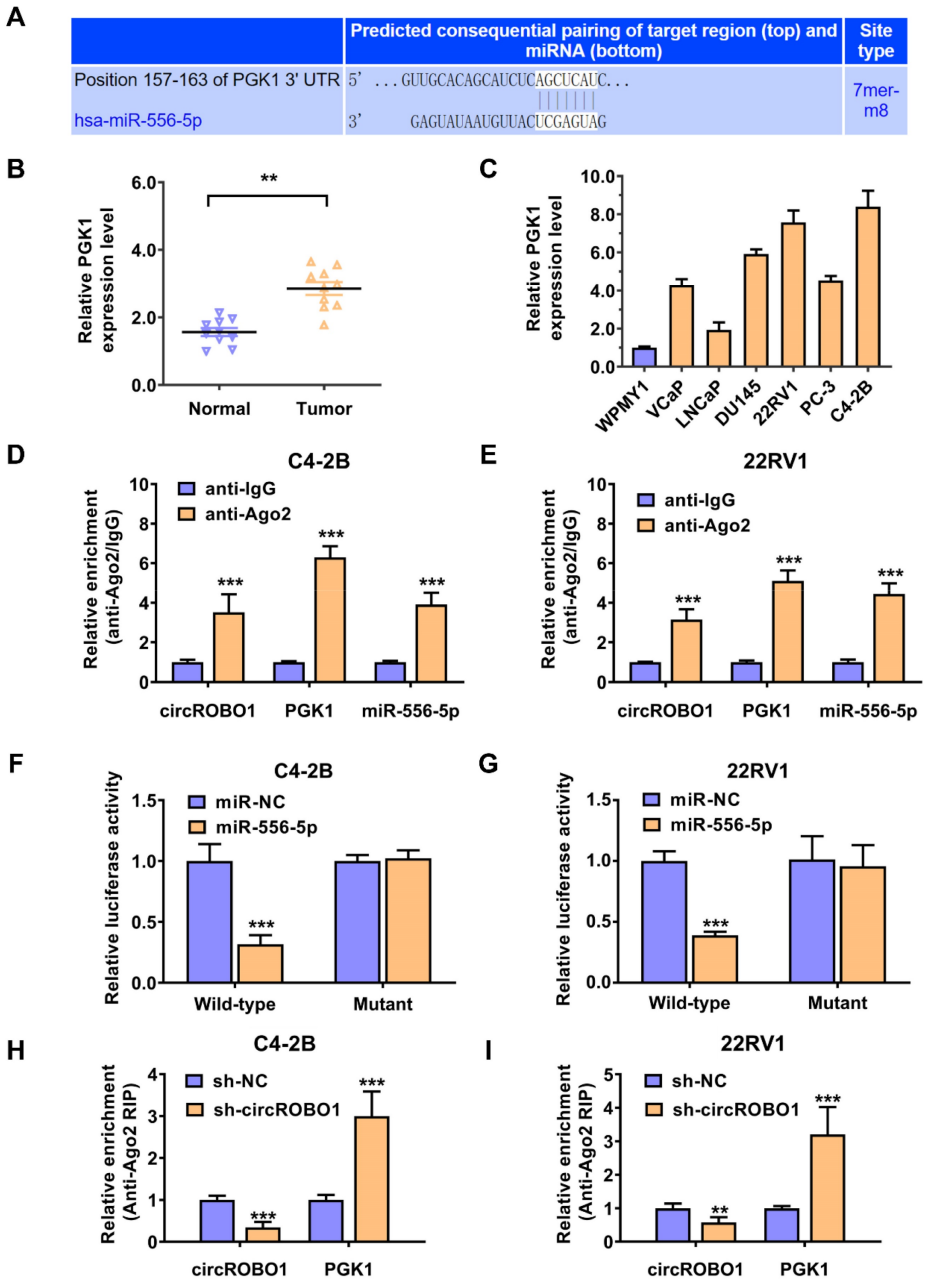
** Glycolysis regulating enzyme PGK1 is the downstream target of miR-556-5p in prostate cancer. (A)** The downstream target of miR-556-5p, PGK1 mRNA, was predicted by TargetScan online. **(B)** Comparison of the expression level of PGK1 in normal WPMY1 cells and prostate cancer cells.** (C)** The expression level of the PGK1 expression level in ten pairs of prostate cancer tissues and adjacent normal tissues, detected by qPCR analysis. **(D-E)** Enrichment of circROBO1, PGK1 and miR-556-5p on AGO2 in C4-2B and 22RV1 prostate cancer cell lines assessed by RIP assay.** (F-G)** Dual luciferase reporter assay of C4-2B and 22RV1 prostate cancer cell lines transfected with miR-556-5p mimics and PGK1 mRNA 3'-UTR wild-type or mutant-type luciferase vectors. **(F)** Enrichment of PGK1 mRNA to AGO2 was significantly increased after the suppression of circROBO1 in C4-2B and 22RV1 prostate cancer cell lines.

To investigate the potential mechanism of miR-556-5p activity, TargetScan was used to analyze the bioinformatics data and discover the potential downstream target of miR-556-5p (http://www.targetscan.org). According to the intersection of TargetScan data and PGK1 binding sites, miR-556-5p putative binding site is conserved in PGK1 mRNA 3'-UTR (Figure 4A). Phosphoglycerate kinase 1 (PGK1) is a glycolytic enzyme that catalyzes the conversion of 1,3-bisphosphoglycerate to 3-phosphoglycerate in the glycolytic pathway 27. PGK1 is not only involved in energy production but also plays a crucial role in regulating tumor progression 28-30. Next, we used RT-qPCR to verify the expression level of the PGK1 expression level in ten pairs of prostate cancer tissues and adjacent normal tissues (Figure 4B). We found that PGK1 was significantly upregulated in tumor tissue compared with their paired normal tissue (Figure 4B). Then, we also detected the expression level of PGK1 in prostate cancer lines using RT-qPCR analysis (Figure 4C). PGK1 was extremely upregulated in prostate cancer cell lines compared to normal cell WPMY1, especially in C4-2B and 22RV1 prostate cancer cell lines, determined by RT-qPCR analysis (Figure 4C). RIP assays were further conducted to verify the molecular mechanism (Figure 4D-E). We found that circROBO1, miR-556-5p, and PGK1 mRNA were all enriched to RNA induced silencing complex in both C4-2B and 22RV1 prostate cancer cells, assessed by the anti-Ago2 related RIP assays (Figure 4D-E). To further confirm the interaction sites between PGK1 mRNA 3'-UTR site and miR-556-5p, we constructed a wild-type and a mutant dual-luciferase reporter plasmids containing the predictive interacting sites between PGK1 mRNA 3'-UTR site and miR-556-5p. Compared to the mutant group, miR-556-5p mimics significantly decreased the luciferase activity of wild-type group. In contrast, there was no detectable difference between the miR-556-5p mimics and control mimics group (Figure 4F-G). Furthermore, both C4-2B and 22RV1 prostate cancer cell lines revealed a decrease in PGK1 mRNA gathering to RISC complexes after targeting circROBO1 (Figure 4H-I).

### circROBO1 promotes prostate cancer enzalutamide resistance and glycolysis through circROBO1-miR-556-5p-PGK1 axis

**Figure 5:**
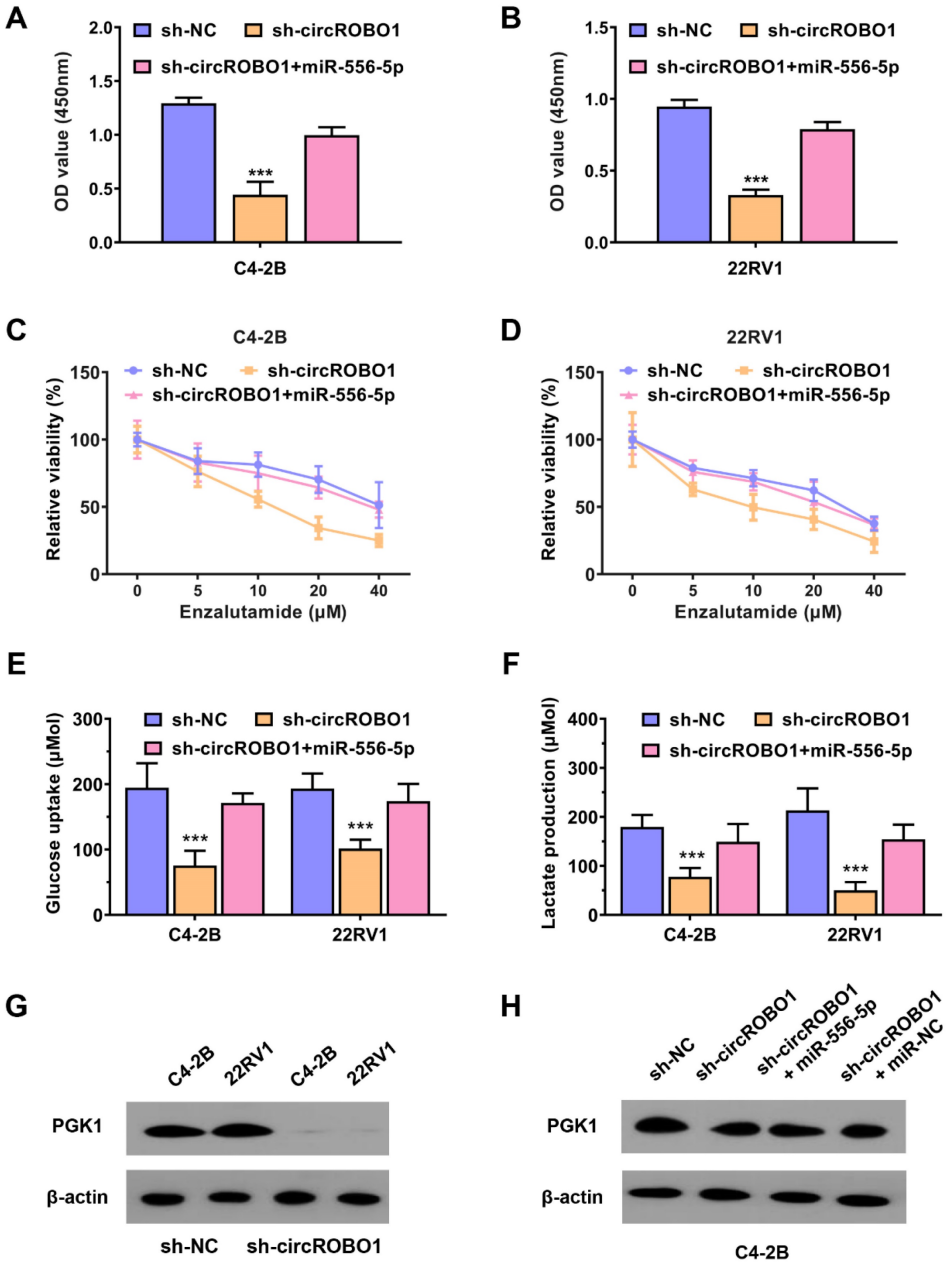
** circROBO1 promotes prostate cancer enzalutamide resistance and glycolysis through circROBO1-miR-556-5p-PGK1 axis. (A-B)** The cell proliferation rate of the C4-2B and 22RV1 prostate cancer cell lines was decreased after the inhibition of circROBO1, which was reversed after the transfection of miR-556-5p mimics. **(C-D)** The enzalutamide resistance was also reversed by the transfection of miR-556-5p mimics when circROBO1 was inhibited in C4-2B and 22RV1 prostate cancer cells. **(E-F)** The glycolysis activity of C4-2B and 22RV1 prostate cancer cells was decreased after silencing of circROBO1, which was also reversed after supplement of miR-556-5p mimics. **(G)** Western blot assay revealed that PGK1 expression was decreased after the suppression of circROBO1 in C4-2B and 22RV1 prostate cancer cell lines. **(H)** Western blot assay revealed that the decrease of PGK1 was rescued via the supplement of miR-335-5p mimics in C4-2B prostate cancer cells, which further determined the circROBO1-miR-556-5p-PGK1 axis in prostate cancer.

To further validate the mechanism of circROBO1-miR-556-5p-PGK1 axis in regulating prostate cancer enzalutamide resistance and glycolysis, we conducted several rescue assays. The proliferation ability of C4-2B and 22RV1 prostate cancer cells was decreased after knocking down of circROBO1, which was reversed after transfection of miR-556-5p mimics (Figure 5A-B). The enzalutamide resistance was also reversed by the transfection of miR-556-5p mimics when circROBO1 was inhibited in C4-2B and 22RV1 prostate cancer cells (Figure 5C-D). The glycolysis activity of C4-2B and 22RV1 prostate cancer cells was decreased after silencing of circROBO1, which was also reversed after supplement of miR-556-5p mimics (Figure 5E-F). PGK1 protein variation in cell lines was determined using Western blot assays. According to our results, PGK1 was extremely decreased after the inhibition of circROBO1 in C4-2B and 22RV1 prostate cancer cells (Figure 5G). This effect could be reversed after supplement of the miR-556-5p mimics, which further determined the circROBO1-miR-556-5p-PGK1 axis in prostate cancer (Figure 5H).

## Discussion

In recent years, circRNAs have become a hot topic in cancer research due to their unique characteristics and potential roles in cancer development and progression 31. circRNAs are highly stable and resistant to RNase degradation compared to linear RNAs, making them more suitable as biomarkers for cancer diagnosis and prognosis 32. circRNAs have been found to regulate gene expression by acting as miRNA sponges, interacting with RNA-binding proteins, and even encoding small peptides 33. These diverse functions of circRNAs provide a new perspective on the complexity of gene regulation in cancer 34. the unique characteristics and diverse functions of circRNAs, as well as their dysregulated expression in cancer, have made them a hot topic in cancer research 35. Although circRNA has been implicated in prostate cancer, its molecular mechanism and biological role have not been well studied.

In the current study, we identified circROBO1 as a significantly downregulated circRNA in both prostate cancer cells and tissues. We then conducted circROBO1 silence experiments to investigate the function of circROBO1 in prostate cancer. The inhibition of circROBO1 significantly decreased the proliferation of prostate cancer cells. In addition, we found that the knockdown of circROBO1 remarkably increased the sensitivity of prostate cancer to enzalutamide treatment. A deceleration in glycolysis rate was observed after inhibition of circROBO1, which could suppress prostate cancer growth and overcome resistance to enzalutamide. Glycolysis is a metabolic pathway that converts glucose into pyruvate, generating ATP and NADH 36. It is a fundamental process in metabolism and is essential for cell survival and proliferation 37-39. However, in cancer cells, glycolysis is often upregulated, even in the presence of oxygen, a phenomenon known as the Warburg effect 40. This metabolic reprogramming is thought to provide cancer cells with the necessary energy and building blocks for rapid proliferation and survival under drug treatment 41. Phosphoglycerate kinase 1 (encoded by PGK1) is a glycolytic enzyme that catalyzes the conversion of 1,3-bisphosphoglycerate to 3-phosphoglycerate in the glycolytic pathway 42. PGK1 is not only involved in energy production but also plays a crucial role in regulating tumor progression 43-46. Our results also showed that knockdown of circROBO1 could decrease the PGK1 expression, which afterwards significantly inhibited the proliferation of cancer cells, and decreased the glucose consumption and lactate production in cancer cells.

According to the theory of competitive endogenous RNA, different types of RNA molecules, including circRNAs, messenger RNAs (mRNAs), long non-coding RNAs (lncRNAs), and pseudogenes, can interact with each other by competing for shared microRNAs (miRNAs). This interaction can lead to changes in gene expression and cellular processes 47. In this research, miR-556-5p was found as a miRNA which could interact with circROBO1 in prostate cancer. miR-556-5p is a recently discovered miRNA that has been shown to play an important role in the progression of various types of cancer. miR-556-5p has been shown to be differentially expressed in various types of cancer. In colorectal cancer, miR-556-5p inhibits cell proliferation and migration by targeting CTBP2, which is involved in the EMT signaling pathway 48. In breast cancer, miR-556-5p inhibits cell proliferation and invasion by targeting YAP1, which is a transcription factor that regulates the expression of genes involved in cell cycle progression and apoptosis 49. In cholangiocarcinoma, miR-556-5p inhibits cell proliferation and migration by targeting YY1, which might be used as a promising therapeutic target for cholangiocarcinoma 50. Understanding the molecular mechanisms of miR-556-5p in regulating prostate cancer progression may provide new insights into the development of novel therapeutic strategies for prostate cancer treatment. Additionally, the findings of our previous study showed that Zoledronic acid enhances cytotoxic T cell response and antitumor immunity in prostate cancer patients 51. Therefore, future study should focus on the function of circROBO1 in the immune evasion of prostate cancer.

In conclusion, our study identified the biological role of circROBO1 in the growth and enzalutamide resistance of prostate cancer through the miR-556-5p-PGK1- glycolysis axis. These findings are important for the development of new treatment strategies and potential prognostic implications for prostate cancer.

## Supplementary Material

Supplementary tables.
