# Supplementary material for: circROBO1 promotes prostate cancer growth and enzalutamide resistance via accelerating glycolysis
Source: J Cancer. 2023 Aug 21;14(13):2574–84. doi: 10.7150/jca.86940 (PMC10475366; doi:10.7150/jca.86940)
Supplement: Supplementary file 1 — Supplementary tables. [file jcav14p2574s1.pdf]

Supplementary Table 1: The detailed clinic parameters of the enrolled patients in this study.

Patients	Age	T stage	N stage	M stage	Gleason score
Patient 1	65	T2	N0	M0	7
Patient 2	62	T4	N1	M0	Unknown
Patient 3	73	T1	N0	M0	7
Patient 4	68	T2	N1	M0	8
Patient 5	72	T3	N1	M0	Unknown
Patient 6	70	T4	N1	M0	9
Patient 7	68	T3	N1	M0	8
Patient 8	75	T3	N0	M0	Unknown
Patient 9	77	T1	N0	M0	Unknown
Patient 10	79	T3	N1	M0	10

Supplementary Table 2: The sequences of the primers used in this study.

Target	Direction	5’ to 3’
circROBO1	F	GCTGGTGACATGGGTTCATACA
circROBO1	R	AAATGGTGGGCTCAGGATGG
ROBO1	F	GGGACCCTATTTCCACTCCC
ROBO1	R	GGGAGCCTGAACAGAGACAT
beta-actin	F	ACAACTTTGGTATCGTGGAAGG
beta-actin	R	GCCATCACGCCACAGTTTC

Supplementary Table 3: The absolute expression levels of circROBO1 between normal and tumor prostate tissues.

	circROBO1-high	circROBO1-low	Statistical analysis
normal tissues	N=1	N=9	* P<0.05
tumor tissues	N=8	N=2	
